# CLINICAL AND LABORATORY CHARACTERISTICS OF SARS-COV-2 INFECTION IN CHILDREN AND ADOLESCENTS

**DOI:** 10.1590/1984-0462/2021/39/2020231

**Published:** 2020-11-16

**Authors:** Marlos Melo Martins, Arnaldo Prata-Barbosa, Maria Clara de Magalhães-Barbosa, Antonio José Ledo Alves da Cunha

**Affiliations:** a Universidade Federal do Rio de Janeiro, Rio de Janeiro, RJ, Brazil.; b Instituto D’Or de Pesquisa e Ensino, Rio de Janeiro, RJ, Brazil.

**Keywords:** SARS-CoV-2, COVID-19, Child, Infant, newborn, Adolescent, SARS-CoV-2, COVID-19, Criança, Recém-nascido, Adolescente

## Abstract

**Objective:**

To present the current evidence on clinical and laboratory characteristics of infection by severe acute respiratory syndrome coronavirus 2 (SARS-CoV-2) during childhood and adolescence.

**Data source:**

This is a narrative review conducted in the databases: Medical Literature Analysis and Retrieval System Online (MEDLINE/PubMed), Latin American and Caribbean Health Sciences Literature in the Virtual Health Library (LILACS/VHL), Scopus, Web of Science, Cochrane Library, portal of the Coordination for the Improvement of Higher Education Personnel (*Coordenação de Aperfeiçoamento de Pessoal de Nível Superior* - CAPES), Scientific Electronic Library Online (SciELO), ScienceDirect, and Cumulative Index to Nursing and Allied Health Literature (CINAHL). The terms used were SARS-CoV-2, COVID-19, novel coronavirus, child, newborn, and adolescent.

**Data synthesis:**

Unlike adults, most children infected by SARS-CoV-2 have mild or asymptomatic clinical presentations. Symptomatic children mainly have low fever and cough, with some associated gastrointestinal symptoms. Severe cases are rare and occur especially in infants under one year of age. Detection of viral particles in feces seems to be more persistent in children and can be used as a tool for diagnosis and control of the quarantine period. Different from adults, children can present distinct inflammatory responses, as has happened in new cases of Kawasaki-like syndrome associated with SARS-CoV-2 infection.

**Conclusions:**

Most children have asymptomatic or mild presentations, with a prevalence of fever, cough, and gastrointestinal symptoms. New cases with different systemic inflammatory reactions in children have been reported, with clinical manifestations distinct from those typically found in adults.

## RESUMO

**Objetivo:**

Apresentar as atuais evidências sobre as características clínicas e laboratoriais da infecção pelo coronavírus da síndrome respiratória aguda grave 2 (SARS-CoV-2) durante a infância e a adolescência.

**Fonte de dados:**

Revisão narrativa realizada nas bases de dados Medical Literature Analysis and Retrieval System Online (MEDLINE/PubMed), Literatura Latino-Americana e do Caribe em Ciências da Saúde na Biblioteca Virtual em Saúde (LILACS/BVS), Scopus, Web of Science, Cochrane Library, portal da Coordenação de Aperfeiçoamento de Pessoal de Nível Superior (CAPES), Scientific Electronic Library Online (SciELO), ScienceDirect e Cumulative Index to Nursing and Allied Health Literature (CINAHL), com o uso dos termos SARS-CoV-2, COVID-19 e novo coronavírus e criança, recém-nascido e adolescente.

**Síntese dos dados:**

Diferentemente dos adultos, as crianças infectadas pelo SARS-CoV-2 apresentam formas clínicas leves ou assintomáticas na maior parte dos casos. As crianças sintomáticas apresentam predominantemente febre baixa e tosse, com alguns sintomas gastrointestinais associados. Casos graves são a minoria e ocorrem especialmente abaixo de um ano de idade. A detecção de partículas virais em fezes parece ser mais persistente em crianças, podendo servir como ferramenta diagnóstica e de controle do tempo de quarentena. Diferentemente dos adultos, as crianças podem apresentar respostas inflamatórias distintas, como tem ocorrido nos novos casos de síndrome de Kawasaki-*like* associada à infecção pelo SARS-CoV-2.

**Conclusões:**

Crianças, na sua maioria, apresentam quadros assintomáticos ou leves, com predomínio de febre, tosse e sintomas gastrointestinais. Novos relatos de diferentes reações sistêmicas inflamatórias em crianças têm sido notados, com manifestações clínicas distintas daquelas tipicamente observadas em adultos.

## INTRODUCTION

Initially detected in the city of Wuhan, a novel coronavirus has gained worldwide prominence for two main characteristics: its highly contagious nature and consequent intercontinental spread and its impact on the global economy and public health.^1^This novel betacoronavirus was called severe acute respiratory syndrome coronavirus 2 (SARS-CoV-2), and the disease it causes was named coronavirus disease 2019 (COVID-19).^2^ The first case series published in China described patients infected by SARS-CoV-2 who progressed to a severe form of pneumonia. Subsequent data showed that approximately 80% of infected people developed a mild clinical presentation, not needing hospitalization, and that 5% required admission to an intensive care unit, with an overall mortality rate of about 5% (https://www.worldometers.info/coronavirus/).^3^

Humans can be infected by SARS-CoV-2 through respiratory droplets or contact with objects contaminated by the virus. During the initial stages of the epidemic, the infection spread to the community mainly through person-to-person contact. At this point, transmission occurred almost exclusively among adults. After this initial phase, in mid-January 2020, the disease also started to be transmitted within family units, spreading to children and older adults.^4^ The first case of infection in children occurred in a family unit, about a week after a trip to the city of Wuhan. It affected a 10-year-old child, who was asymptomatic but had ground-glass opacities on the chest computed tomography (CT).^5^

Since then, case reports, case series, and small cohort studies have described the clinical and laboratory characteristics of COVID-19 in children. Generally, the vast majority of infected children have asymptomatic or mild cases of the disease, unlike adults.^6^ Even during recent epidemics of severe acute respiratory syndrome coronavirus (SARS-CoV) in Hong Kong and Middle East respiratory syndrome coronavirus (MERS-CoV) in South Korea, few pediatric patients have been reported.^7^ ^,^ ^8^

This review aimed to present the current evidence on clinical and laboratory characteristics of SARS-CoV-2 infection during childhood and adolescence.

## METHOD

This is a narrative review conducted in the databases: Medical Literature Analysis and Retrieval System Online (MEDLINE/PubMed), Latin American and Caribbean Health Sciences Literature in the Virtual Health Library (LILACS/VHL), Scopus, Web of Science, Cochrane Library, portal of the Coordination for the Improvement of Higher Education Personnel (*Coordenação de Aperfeiçoamento de Pessoal de Nível Superior* - CAPES), Scientific Electronic Library Online (SciELO), ScienceDirect, and Cumulative Index to Nursing and Allied Health Literature (CINAHL). We used the following strategy: (“COVID-19” OR “severe acute respiratory syndrome coronavirus 2” OR “2019 novel coronavirus infection” OR “COVID19” OR “coronavirus disease 2019” OR “coronavirus disease-19” OR “2019-nCoV disease” OR “2019 novel coronavirus disease” OR “2019-nCoV infection” OR “Wuhan coronavirus” OR “Wuhan seafood Market pneumonia virus” OR “COVID19 virus” OR “COVID-19 virus” OR “coronavirus disease 2019 virus” OR “SARS-CoV-2” OR “SARS2” OR “2019-nCoV” OR “2019 novel coronavirus” OR “novel coronavirus” OR “new coronavirus”) AND (“Child” OR “Children” OR “Minors” OR “Infant” OR “Newborn” OR “Neonate” OR “neonatal” OR “adolescent” OR “adolescence” OR “teen” OR “teenager” OR “youth”). After careful evaluation, we selected articles addressing the clinical characteristics of SARS-CoV-2 infection in the neonatal period, childhood, and/or adolescence, based on their association with the proposed theme.

**Figure 1:**
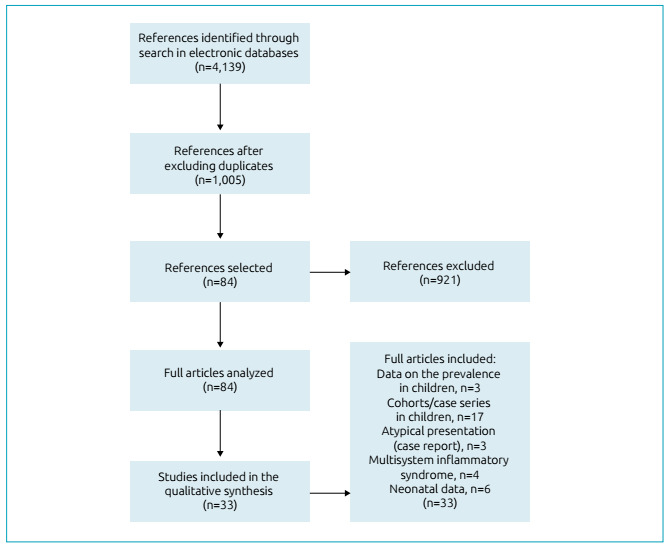
Article selection flowchart for this narrative review.

The inclusion criteria were case reports, case series, or cohort studies that described clinical and laboratory characteristics of COVID-19 in children. The exclusion criteria were articles published in a language other than English, Portuguese, Spanish, or French. The literature search occurred in June 2020. Initially, we found 4,139 references. After excluding duplicates, an initial screening based on the title, and reading the full text, 33 articles remained. Figure 1 presents the article selection flowchart for this review.

### Clinical characteristics of SARS-CoV-2 infection in children and adolescents

Few epidemiological data have been published on the prevalence of SARS-CoV-2 infection in children. According to data from the World Health Organization (WHO)-China Joint Mission on COVID-19, 55,924 cases had been laboratory-confirmed in China until February 20, 2020, of which 2.4% corresponded to individuals under 19 years of age. Among them, 2.5% had a severe manifestation of the disease, and 0.2% had a critical presentation.^9^ The Centers for Disease Control and Prevention (CDC) recently published United States data on SARS-CoV-2 infection in children: 149,760 laboratory-confirmed cases occurring between February 12 and April 2, 2020 were analyzed, and, among the 149,082 (99.6%) cases with age information, 2,572 (1.7%) corresponded to individuals younger than 18 years, with 5.7% of them requiring hospitalization and only 3 reported deaths.^10^Dong et al.^11^ analyzed 2,143 COVID-19 cases in children reported to the Chinese Center for Disease Control and Prevention from January 6 to February 8, 2020: 731 (34.1%) had laboratory confirmation, and 1,412 (65.9%) were suspected cases, with a mean age of 7 years (interquartile range 2-13 years). Among all cases (confirmed and suspected), 94 (4.4%) were asymptomatic, 1,091 (50.9%) were mild, and 831 (38.8%) were moderate cases. The proportion of severe and critical cases was inversely proportional to the different age groups: <1 year (10.6%), 1-5 years (7.3%), 6-10 years (4.2%), 11-15 years (4.1%), and ≥16 years (3%).

After publication of the first COVID-19 case in a 10-year-old child in Shenzhen, China, with an asymptomatic presentation, despite the finding of ground-glass opacity on the chest CT, several case reports and small cohort studies of children began to be published.^5^ ^,^ ^12^ ^,^ ^13^ ^,^ ^14^ ^,^ ^15^ ^,^ ^16^ ^,^ ^17^ ^,^ ^18^ ^,^ ^19^ ^,^ ^20^ ^,^ ^21^ ^,^ ^22^ ^,^ ^23^ ^,^ ^24^ ^,^ ^25^ ^,^ ^26^ ^,^ ^27^ ^,^ ^28^ Tables 1, 2, and 3 summarize the main clinical, laboratory, and imaging findings, as well as information about the need for oxygen therapy from cohort studies and case series involving children and adolescents.

**Table 1:** Summary of clinical, laboratory, and imaging characteristics, as well as information about the need for oxygen therapy.

	Han et al.^12^	Xia et al.^13^	Zhu et al.^21^	Qiu et al.^22^	Zheng et al.^23^	Lu et al.^24^
Number of children	7	20	10	36	25	171
Age group (years)	0-13	0-14	1-18	1-16	0-14	0-15
Male/female	4/3	13/7	5/5	23/13	14/11	104/67
Location	China	China	China	China	China	China
Period	January February	January February	January February	January March	February	January February
Fever	5 (71.4%)	12 (60%)	4 (40%)	13 (36.1%)	13 (52%)	71 (41.5%)
Cough	5 (71.4%)	13 (65%)	3 (30%)	7 (19.4%)	11 (44%)	83 (48.5%)
Myalgia/fatigue	0	1 (5%)	---	---	---	13 (7.6%)
Diarrhea, nausea, and/or vomiting	4 (57.1%)	3 (15%)	0	---	3 (12%)	15 (8.8%)
Odynophagia	1 (14.3%)	1 (5%)	0	---	---	---
Dyspnea/ tachypnea	3 (42.9%)	2 (10%)	0	1 (2.8%)	2 (8%)	49 (28.7%)
Abnormality on chest CT	5 (71.4%)	16 (80%)	5 (50%)	19 (52.8%)	17 (68%)	111 (64.9%)
Leukocytosis	2 (28.6%)	2 (10%)	---	---	---	---
Neutropenia	1 (14.3%)	---	---	---	---	---
Lymphocytosis	---	3 (15%)	---	---	---	---
Lymphopenia	---	7 (35%)	0	11 (30.6%)	10 (40%)	6 (3.5%)
Leukopenia	---	4 (20%)	0	7 (19.4%)	---	---
Increased AST and ALT	2 (28.6%)	5 (25%)	3 (30%)	2 (5.6%)	---	---
Increased CK/CK-MB	4 (57.1%)	15 (75%)	---	11 (30.6%)	---	---
Increased CRP	2 (28.6%)	16 (80%)	0	1 (2.8%)	---	---
Increased procalcitonin	3 (42.9%)	16 (80%)	0	6 (16.7%)	---	---
Increased ESR	3 (42.9%)	---	---	---	---	---
Need for oxygen	2 (28.6%)	---	1 (10%)	6 (16.7%)	2 (8%)	4 (2.3%)

CT: computed tomography; AST: aspartate transaminase; ALT: alanine transaminase; CK: creatine kinase: CK-MB: CK MB isoenzyme; CRP: C-reactive protein; ESR: erythrocyte sedimentation rate.

**Table 2:** Summary of clinical, laboratory, and imaging characteristics, as well as information about the need for oxygen therapy.

	Xu et al.^25^	Cai et al.^26^	Su et al.^27^	Du et al.^28^	Tan et al.^14^	Garazzino et al.^15^
Number of children	10	10	9	14	10	168
Age group (years)	0-15	0-10	0-9	0-16	1-12	0-17
Male/female	5/5	4/6	3/6	6/8	3/7	94/74
Location	China	China	China	China	China	Italy
Period	January February	January February	January February	January February	January March	January March
Fever	7 (70%)	8 (80%)	2 (22.2%)	5 (35.7%)	4 (40%)	138 (82.1%)
Cough	5 (50%)	6 (60%)	1 (11.1%)	3 (21.4%)	3 (30%)	82 (48.8%)
Myalgia/fatigue	---	---	---	---		3 (1.8%)
Diarrhea, nausea, and/or vomiting	3 (30%)	0	---	---	1 (10%)	22 (13.1%)
Odynophagia	4 (40%)	4 (40%)	---	---	---	9 (5.4%)
Dyspnea/tachypnea	---	0	---	---	---	16 (9.5%)
Abnormality on chest CT	5 (50%)	4 (40%)	---	6 (42.8%)	5 (50%)	---
Leukocytosis	---	3 (30%)	---	---	1 (10%)	---
Neutropenia	2 (20%)	3 (30%)	---	---	0	---
Lymphocytosis	3 (30%)	1 (10%)	---	---	0	---
Lymphopenia	3 (30%)	0	---	---	0	---
Leukopenia	1 (30%)	1 (10%)	---	---	0	---
Increased AST and ALT	1 (10%)	2 (20%)	0	---	---	---
Increased CK/CK-MB	0	5 (50%)	6 (66.6%)	---	---	---
Increased CRP	3 (30%)	3 (30%)	0	---	---	47/121 (38.8%)
Increased procalcitonin	5 (50%)	0	---	---	---	---
Increased ESR	3 (30%)	---	0	---	---	---
Need for oxygen	0	0	0	0	0	---

CT: computed tomography; AST: aspartate transaminase; ALT: alanine transaminase; CK: creatine kinase: CK-MB: CK MB isoenzyme; CRP: C-reactive protein; ESR: erythrocyte sedimentation rate.

**Table 3:** Summary of clinical, laboratory, and imaging characteristics, as well as information about the need for oxygen therapy.

	Ma et al.^16^	Sun et al.^17^*	Liu et al.^18^	Zhong et al.^19^	Dodi et al.^20^
Number of children	115	8	4	9	14
Age group (years)	---	0-15	0-9	0-12	---
Male/female	73/42	6/2	2/2	4/5	9/5
Location	China	China	China	China	Italy
Period	---	January February	---	---	January April
Fever	29 (25.2%)	6 (75%)	3 (75%)	2 (22.2%)	14 (100%)
Cough	47 (40.9%)	6 (75%)	3 (75%)	5 (55.5%)	5 (35.7%)
Myalgia/fatigue	---	1 (12.5%)	1 (25%)		3 (21.4%)
Diarrhea, nausea, and/or vomiting	---	4 (50%)	---	0	2 (14.3%)
Odynophagia	---	---	---	---	7 (50%)
Dyspnea/tachypnea	---	8 (100%)	---	---	---
Abnormality on chest CT	49 (42.6%)	8 (100%)	3 (75%)	---	---
Leukocytosis	---	---	0	1 (11.1%)	---
Neutropenia	---	---	2 (50%)	5 (55.5%)	---
Lymphocytosis	---	---	2 (50%)	2 (22.2%)	---
Lymphopenia	---	---		1 (11.1%)	1 (7.1%)
Leukopenia	---	---	1 (25%)	1 (11.1%)	---
Increased AST and ALT	11 (9.6%)	4 (50%)		---	---
Increased CK/CK-MB	34 (29.6%)	---		---	---
Increased CRP	---	5 (62.5%)	1 (25%)	9 (100%)	---
Increased procalcitonin	---	5 (62.5%)		0	---
Increased ESR	---	---		---	---
Need for oxygen	3 (2.6%)	8 (100%)*	0	0	0

CT: computed tomography; AST: aspartate transaminase; ALT: alanine transaminase; CK: creatine kinase: CK-MB: CK MB isoenzyme; CRP: C-reactive protein; ESR: erythrocyte sedimentation rate.

*Study only analyzed children with a severe presentation of COVID-19.

In the studies analyzed, symptomatic children showed a predominance of fever (22.2-100%) and cough (11.1-75%), with some associated gastrointestinal symptoms, including nausea, vomiting, diarrhea, and abdominal pain (8.8-57.1%). Many children presented abnormalities in lung imaging tests (40-100%), even though most of them had mild cases. Increases in serum levels of creatine kinase MB isoenzyme (CK-MB), C-reactive protein (CRP), and procalcitonin were found in a large number of these children. Less frequent laboratory findings included leukocytosis, leukopenia, lymphopenia, lymphocytosis, neutropenia, and increased transaminase levels and erythrocyte sedimentation rate (ESR). The need for oxygen was low (2.3-28.6%), except in the study by Sun et al.,^17^ who described only severe cases in children.

Some studies compared these characteristics with those of adults. Han et al.^12^ reported that children showed a higher frequency of nausea and/or vomiting, leukocytosis, and increased serum CK compared to adults. Xia et al.^13^ found higher levels of procalcitonin in children. Du et al.^28^ identified a prevalence of cough and phlegm in children compared to adults. Xu et al.^25^ described the persistence of positivity in the reverse transcription-polymerase chain reaction (RT-PCR) for SARS-CoV-2 on rectal swabs collected from children, even after negative PCR on nasopharyngeal samples. Two patients persisted with positive results in their feces for 13 and 20 days. Cai et al.^26^ also described this persistence for up to 30 days and after negative nasopharyngeal swab PCR. Su et al.^27^ reported the same finding when analyzing nine children from the Jihan region, in China.

While studying eight severe COVID-19 cases in children, Sun et al.^17^ found a predominance of tachypnea (100% of patients), and fever with cough in six of the children investigated. Increased CRP, procalcitonin, lactate dehydrogenase (LDH), transaminase, and d-dimer levels were also detected. Two patients required mechanical ventilation, and the other six needed high flow oxygen. No death was reported.

Some case reports included less common manifestations in children infected by SARS-CoV-2. Paret et al.^29^ described two children aged 25 and 56 days with confirmed SARS-CoV-2 infection, presenting with fever without respiratory symptoms. Besides fever, the 25-day-old newborn had rash and irritability, and the parents were symptomatic at the time. The second child presented only fever, without other associated symptoms, and the parents were asymptomatic. Wu et al.^30^ described a child aged 2 years and 10 months with conjunctivitis, eyelid dermatitis, normal chest CT, increased LDH and CK-MB, lymphocytosis, and neutropenia, with positive nasopharyngeal swab PCR for SARS-CoV-2*.* Seizures have also been reported in children infected by SARS-CoV-2. Tan et al.^14^ found a patient who had seizures in the Hunan province in China. Garazzino et al.^15^ identified non-febrile episodes in 3 children (1.8%), and febrile episodes in 2 (1.2%) out of the 168 studied. Dugue et al.^31^ also described a six-week-old infant with an initial case of fever and cough, presenting episodes of sustained upward gaze with tonic lower limb posturing, as well as electroencephalogram with temporal sharp waves, normal brain magnetic resonance imaging, and positive PCR for SARS-CoV-2 on nasopharyngeal swab and feces and negative on blood and cerebrospinal fluid samples.

### Multisystem inflammatory syndrome possibly related to SARS-CoV-2 infection

Recently, in the state of New York, United States, 166 children developed multisystem inflammatory syndrome, possibly related to SARS-CoV-2 infection.^32^ The first cases were reported about a month after the emergence of COVID-19 cases in the area, suggesting an initial post-infectious immune response. The syndrome is characterized by persistent fever and features of Kawasaki syndrome, in addition to toxic shock syndrome. School-aged children were the most affected, with three deaths reported. Many of these children were admitted to intensive care units for cardiac and respiratory support, and most cases tested positive for SARS-CoV-2 and its specific antibodies.^33^

Riphagen et al.^34^ reported clinical and laboratory characteristics of eight children diagnosed with hyperinflammatory shock, similar to atypical Kawasaki disease and toxic shock syndrome, admitted to the South Thames Retrieval Service in London, UK. The authors highlighted the very high number of children with this condition over a short period (10 days) during the second half of April 2020. The clinical presentation was very similar among these children - fever between 38 and 40^o^C, rash, conjunctivitis, peripheral edema, limb pain, in addition to gastrointestinal symptoms. All children progressed to vasoplegic shock, refractory to volume resuscitation, occasionally requiring noradrenaline and milrinone. None of these patients had respiratory symptoms, despite seven of them needing mechanical ventilation for cardiovascular stabilization. Other findings included pericardial and pleural effusions and ascites, denoting a diffuse inflammatory process. Two of these children tested positive for SARS-CoV-2, including the only one who died, and other three children had negative results but with a family history of suspected or confirmed COVID-19. Adenovirus was isolated in only one child. The authors suggest the emergence of a new phenomenon of hyperinflammatory syndrome possibly related to previous or recent asymptomatic SARS-CoV-2 infection in children.

Verdoni et al.^35^ also observed a 30-fold increase in the number of cases of Kawasaki disease in the region of Bergamo, Italy, during April 2020, coinciding with the SARS-CoV-2 epidemic period. The authors conducted a study comparing children with Kawasaki disease before (19 patients) and after (10 patients) the SARS-CoV-2 epidemic in the same region. They concluded that the two groups (before group vs. after group) differed regarding the incidence of the disease - 0.3 vs. 10 cases per month; mean age - 3 vs. 7.5 years; cardiac involvement - 2 of 19 vs. 6 of 10; incidence of Kawasaki disease shock syndrome - 0 of 19 vs. 5 of 10; incidence of macrophage activation syndrome - 0 of 19 vs. 5 of 10; and need for corticosteroid therapy - 3 of 19 vs. 8 of 10. SARS-CoV-2 antibodies were found in 8 of the 10 patients of the second group.

Based on the above findings, Table 4 presents the preliminary criteria for defining this inflammatory syndrome.

**Table 4:** Preliminary criteria for defining cases of “multisystem inflammatory syndrome in children and adolescents temporally related to coronavirus disease 2019 (COVID-19),” according to the World Health Organization.^43^

Children and adolescents aged 0 to 19 years with fever for 3 days or longer
**AND** Elevated inflammatory markers, such as ESR, CRP, or procalcitonin
**AND** No other cause for microbial inflammation, including bacterial sepsis and staphylococcal and streptococcal toxic shock syndromes
**AND** Evidence of COVID-19 (RT-PCR, antigen test, or positive serology) or likely contact with a COVID-19 patient
**AND** two of the following criteria: 1. rash, bilateral non-purulent conjunctivitis, or signs of mucocutaneous inflammation (oral, hands, or feet); 2. hypotension or shock; 3. characteristics of myocardial dysfunction, pericarditis, valvulitis, or coronary abnormalities (including echocardiogram findings or elevated troponin/pro-brain natriuretic peptide); 4. evidence of coagulopathy; 5. acute gastrointestinal problems (diarrhea, vomiting, or abdominal pain).

ESR: erythrocyte sedimentation rate; CRP: C-reactive protein; RT-PCR: reverse transcription polymerase chain reaction.

### Clinical characteristics of SARS-CoV-2 infection in the neonatal period

The evidence available is not enough to confirm the vertical transmission of SARS-CoV-2. Case reports and case series have been published describing the clinical characteristics of newborns of mothers with confirmed SARS-CoV-2 infection.

Zeng et al.^36^ identified 33 newborns whose mothers had COVID-19 in Wuhan Children’s Hospital, and only three of these babies had clinical manifestations. The first had fever and lethargy in the second day of life, radiological signs of pneumonia on the chest X-ray, and increased procalcitonin, in addition to positive nasopharyngeal and rectal swab PCR for SARS-CoV-2 in the second and fourth days of life. The second case presented fever, vomiting, and lethargy, as well as leukocytosis, lymphopenia, increased CK-MB, pneumonia on the chest X-ray, and positive nasopharyngeal and rectal swab PCR for SARS-CoV-2 in the second and fourth days of life. The third child was born prematurely (31 weeks and two days), progressing to perinatal asphyxia, pneumonia, and sepsis, also with positive rectal and nasopharyngeal swab for SARS-CoV-2 until the fourth day of life.

Zhang et al.^37^ retrospectively reported all laboratory-confirmed COVID-19 cases in China, registered in the National Health Commission. Out of the 81,026 cases reported until March 13, 2020, they identified 4 newborns (<28 days of life) infected by SARS-CoV-2, aged from 30 hours to 17 days. All were hospitalized. Two patients presented with fever, one with tachypnea, one had a cough, and one remained asymptomatic. Diagnosis was made by nasopharyngeal swab PCR in two newborns and rectal swab in the others. Chest CT was performed in three patients, showing an increase in vascular markings. None of the newborns required oxygen support or presented complications of the disease. Three mothers infected by SARS-CoV-2 had symptoms before delivery and one after. The most common maternal symptoms were fever, cough, and loss of appetite. Three of these neonates were born by cesarean delivery at biosafety level III (personal protection equipment to prevent contamination by infectious agents transmitted by aerosols and that can cause severe diseases), were separated from their mothers right after birth, and were not breastfed.

Dong et al.^38^ described a potential case of vertical transmission in a pregnant woman with COVID-19, diagnosed in the 34^th^ week of gestation. The baby was born at term by cesarean delivery in a negative-pressure isolation room, and the pregnant woman used an N95 mask. Immediately after birth, the newborn was isolated from the mother, did not receive breast milk, remaining asymptomatic throughout the period. Immunoglobulin M and G (IgM and IgG) were positive for SARS-CoV-2 in a sample collected from the newborn two hours after birth. The child also presented high levels of serum cytokines and leukocytosis. Chest CT scan was normal. Nasopharyngeal swab PCR for SARS-CoV-2 was performed at five different moments between two hours and 16 days of life, all negative. Serum IgM and IgG for SARS-CoV-2 were still detectable on the 15^th^ day of life.

Wang et al.^39^ reported the case of a 19-day-old newborn, with fever, vomiting, refusing to eat, with increased frequency of defecation, and no apparent respiratory symptoms. The parents were infected by SARS-CoV-2 and symptomatic, and the newborn tested positive for SARS-CoV-2 on material collected from both nasopharyngeal and rectal swabs. Late sepsis has also been reported in newborns infected by SARS-CoV-2.^40^ ^,^ ^41^

## DISCUSSION

Unlike adults, children infected by SARS-CoV-2 have mild or asymptomatic clinical presentations, according to most cases described in the literature. The child’s condition of asymptomatic virus carrier probably has a great impact on the forms of community transmission, as the identification of asymptomatic carriers is a great challenge around the world. According to recent studies, symptomatic children tend to have low fever and cough, with some associated gastrointestinal symptoms, including nausea, vomiting, diarrhea, and abdominal pain, with a good recovery between one and two weeks. Severe cases have been reported, but they are few and apparently more prevalent in infants under one year of age.

The prevalence of gastrointestinal symptoms in some children, associated with the persistence of the virus in fecal samples, even after negative nasopharyngeal swabs, suggests that the gastrointestinal tract might be a site of viral replication in this age group, in addition to representing an important form of interpersonal transmission. Although the clinical significance of more persistent viral particles in children’s feces is not yet clear, the use of nasopharyngeal and fecal samples could result in greater sensitivity in detecting the virus in suspected children or those who have had contact with confirmed cases, acting both as an important diagnostic tool and as a way of controlling the quarantine period.

The seemingly higher prevalence of increased serum levels of CK-MB and procalcitonin might represent an inflammatory response to SARS-CoV-2. CK-MB is an indicator of myocardial injury, indicating the possible role of the virus in cardiac lesions. Different from adults, children may present different inflammatory responses and, therefore, distinct clinical repercussions, as has occurred in New York State, England, and Italy, with cases of multisystem inflammatory syndrome/Kawasaki disease associated with SARS-CoV-2 infection. In contrast, published studies revealed that many children presented abnormalities in lung imaging tests, even though most of them had asymptomatic and mild cases.

The current SARS-CoV-2 pandemic has been associated with a higher incidence of severe cases of Kawasaki-like disease in children from different regions of the world. The pathophysiology of these presentations is still unknown but may be related to the cytokine storm detected in severe manifestations of COVID-19 in adults.^42^ Table 4 shows the preliminary criteria for defining cases of “multisystem inflammatory syndrome in children and adolescents temporarily related to COVID-19,” according to WHO, based on clinical and laboratory characteristics of published cases.^43^

Current evidence shows low rates of peripartum transmission of SARS-CoV-2 and is inconclusive concerning intrauterine transmission. Newborns can be infected by the virus after birth and, theoretically, represent a risk group due to their still immature immune system. Researchers believe that the main form of COVID-19 transmission to newborns is through droplets of infected caregivers or contact with contaminated material. Therefore, care must focus on two main pillars: avoiding infection of the newborn and of health professionals in the delivery room by adopting preventive measures related to infection by droplets or contact.

The reasons for most children infected by SARS-CoV-2 presenting asymptomatic or mild cases are still not understood. Some speculations are made in the literature:

- Compared to adults, children have a smaller range of activities; therefore, they are primarily infected in their family unit. As in other viruses, SARS-CoV-2 viral ribonucleic acid (RNA) is subject to replication errors and mutations, reducing its virulence. Thus, children could be more frequently infected by a second- or third-generation virus, leading to milder cases.
- Children may have a different immune response to SARS-CoV-2 compared to adults. The innate immune system, responsible for the early response to pathogens, seems to be more developed in children than in adults. The adaptive immune system, which learns to recognize pathogens, seems to be more prevalent in adults, leading to a slightly later response. Other coronaviruses, such as SARS and MERS, showed this same pattern of immune system response.^44^
- Adults are potentially more exposed to different viral infections and, consequently, might have produced antibodies against viral antigens on a larger scale, resulting in a cross-reaction with SARS-CoV-2 and triggering a more exuberant inflammatory response.^45^
- Another immunologic possibility is the antibody-dependent enhancement mechanism, as occurs with the dengue virus.^45^
- Recent evidence suggests that the angiotensin-converting enzyme 2 (ACE2) cellular receptor and the transmembrane protease serine 2 (TMPRSS2), necessary for SARS-CoV-2 to enter cells and be distributed to different organic tissues, may be different in children and adults. In children, ACE2 receptors can present a different structure, concentration, or ability to connect with the virus.^46^ ^,^ ^47^
- Children have a larger number of other viruses in the lung and airway mucosa, which could limit the replication of SARS-CoV-2 by direct virus-to-virus competition.^48^
- The pediatric population is less prone to develop severe acute respiratory syndrome (SARS) in viral respiratory tract infections than adults.^49^

Most children infected by SARS-CoV-2 have asymptomatic or mild presentations, with a prevalence of fever, cough, and gastrointestinal symptoms. Children can have severe cases of the disease, although the risk seems to be lower when compared to adults. Currently, one of the main challenges is efficiently identifying these oligosymptomatic or asymptomatic children because they might represent an important source of interpersonal transmission. New cases with different systemic inflammatory reactions in children have been reported, with clinical manifestations distinct from those typically found in adults. Apparently, peripartum transmission rates of SARS-CoV-2 are low and intrauterine transmission remains unproven. Neonatal care must focus on preventing postnatal transmission from the infected mother and relatives to the newborn.
